# Renal telocytes contribute to the repair of ischemically injured renal tubules

**DOI:** 10.1111/jcmm.12274

**Published:** 2014-04-24

**Authors:** Liping Li, Miao Lin, Long Li, Rulin Wang, Chao Zhang, Guisheng Qi, Ming Xu, Ruiming Rong, Tongyu Zhu

**Affiliations:** a Department of Urology, Fudan University Zhongshan HospitalShanghai, China; b Shanghai Key Lab of Organ TransplantationShanghai, China

**Keywords:** Telocyte, ischaemia–reperfusion, kidney, organ regeneration, inflammation, apoptosis

## Abstract

Telocytes (TCs), a distinct type of interstitial cells, have been identified in many organs *via* electron microscopy. However, their precise function in organ regeneration remains unknown. This study investigated the paracrine effect of renal TCs on renal tubular epithelial cells (TECs) *in vitro*, the regenerative function of renal TCs in renal tubules after ischaemia–reperfusion injury (IRI) *in vivo* and the possible mechanisms involved. In a renal IRI model, transplantation of renal TCs was found to decrease serum creatinine and blood urea nitrogen (BUN) levels, while renal fibroblasts exerted no such effect. The results of histological injury assessments and the expression levels of cleaved caspase-3 were consistent with a change in kidney function. Our data suggest that the protective effect of TCs against IRI occurs *via* inflammation-independent mechanisms *in vivo*. Furthermore, we found that renal TCs could not directly promote the proliferation and anti-apoptosis properties of TECs *in vitro*. TCs did not display any advantage in paracrine growth factor secretion *in vitro* compared with renal fibroblasts. These data indicate that renal TCs protect against renal IRI *via* an inflammation-independent pathway and that growth factors play a significant role in this mechanism. Renal TCs may protect TECs in certain microenvironments while interacting with other cells.

## Introduction

Acute renal failure (ARF), primarily caused by renal ischaemia–reperfusion injury (IRI) following episodes of hypotension or surgical cross-clamping of the aorta and/or renal arteries, is a common disease with high morbidity and mortality. Ischaemia–reperfusion injury leads to alterations in cell metabolism, inflammation and free radical generation, resulting in apoptosis of renal tubular epithelial cells (TECs) [1]. Traditional treatments for ARF have led to limited improvement [2]. Several therapies, including the use of growth factors and stem cells, have been demonstrated to effectively ameliorate ARF in various animal models. However, all of these therapies have failed in clinical practice [3,4]. Therefore, novel strategies are required for the treatment of ARF.

Telocytes (TCs), a distinct type of interstitial cells, were recently identified. Telocytes were found in heart [5–12], in lungs [13–16] as well as in kidneys [17,18]. The presence of TCs was also reported in many other tissues and organs [5,19–36]. Microarray-based gene expression analysis and microRNA signature have been used to distinguish TCs from other interstitial cell types [37,38], mainly fibroblasts. Genetically, the expression of more than 1000 genes has been found to be up- or down-regulated, respectively, compared to fibroblasts. Morphologically, TCs exhibit a small cell body and long, thin prolongations, termed telopodes (Tps; generally, 2–3/cell; length of 10–100 μm). Therefore, TCs have been deemed a unique cell type, distinct from fibroblasts, because of their unique ultrastructure and immunocytochemical profile [39].

Functionally, TCs have been reported to develop a 3D network through the organ interstitium that communicates with surrounding organ-specific structures, immune cells, nerve endings and even stem cells [6,8,9,11,12]. Moreover, TCs may play an assisting role in facilitating the regenerative function of stem cells in stem cell niches [6,12,13,30,33,36]. In the gene expression profile of TCs, collagen type IV, tissue inhibitor of metalloproteinases 1 and 3 (TIMP1 and TIMP3), and matrix metallopeptidases 3 and 10 (MMP3 and MMP10) are highly expressed, all of which are components or regulators of the tubular basement membrane [40]. Recent studies have revealed that cardiac TCs can facilitate the functional regeneration of the heart following myocardial infarction in an animal model [10].

In the present study, we first demonstrated the existence of TCs in the human kidney cortex *via* electron microscopy and immunocytochemistry and subsequently generated primarily cultures of TCs from the human kidney *in vitro* [17]. We further investigated the paracrine effect of renal TCs on renal TECs *in vitro*, the regenerative function of renal TCs on the renal tubule after IRI *in vivo* and the potential mechanisms of these effects.

## Materials and methods

### Animals and study design

Three-month-old male Sprague–Dawley (SD; 150–200 g) rats were utilized in this study. The rats were housed with food and water for at least 2 weeks prior to experimentation. The rats were separated into five groups: a normal control group (*n* = 5) in which the rats were not subjected to operation; a normal sham control group (*n* = 5) in which the rats were subjected to sham operation without clamping the renal arteries; a PBS-injected group (*n* = 5) in which the rats were subjected to operation with PBS injection; a fibroblast-injected group (*n* = 5) in which the rats were subjected to operation with fibroblast injection; and a TC-injected group (*n* = 5) in which the rats were subjected to operation with TC injection. All of the animal experiments described here were performed in accordance with the guidelines of the Ministry of Science and Technology of the People's Republic of China [(2006)398] and were approved by the Fudan University Animal Care and Use Committee.

### Cell culture

Renal TCs were isolated and primary cultures were generated as previously described [27]. Briefly, we obtained sterile samples from SD rats. The samples were minced into small pieces with a volume of ∽1 mm^3^ and washed three times with phosphate-buffered saline. Then, the samples were digested by using 10 mg/ml collagenase type II (Sigma-Aldrich, St. Louis, MO, USA) and 2000 U/ml deoxyribonuclease I (Sigma-Aldrich) in PBS without Ca2+ or Mg2+ for 4 hrs on an orbital shaker at 37°C. The dispersed cells were collected *via* centrifugation at 284 × g and separated *via* filtration through 40 μm-diameter cell strainers. The cells were cultured in DMEM/F12 medium (Gibco, Portland, OR, USA) supplemented with 10% Fetal Bovine Serum (FBS). The medium was changed every 48 hrs, and phase-contrast microscopy (Olympus 1X51; Olympus Corporation, Tokyo, Japan) was performed to monitor the growth of the TCs. Renal fibroblasts were purchased from the Aiyan Biological Research Company (Shanghai, China) and were cultured in high-glucose DMEM (Gibco) supplemented with 10% FBS. A rat renal TEC line (NRK-52E) was obtained from the Shanghai Branch of the Chinese Academy of Science and was cultured in high-glucose DMEM (Gibco) supplemented with 5% FBS.

### Purity of the isolated renal TCs

The purity of the isolated renal TCs was determined *via* double immunofluorescence staining for CD117 and CD34. Isolated renal TCs (104) were cultured on a coverslip and fixed by using ice-cold methanol for 15 min. After washing three times with PBS (pH = 7.4), a rabbit anti-rat CD117 antibody (1:300; cat. no. NBP1-19865; Novus, Littleton, CO, USA) was added, and the samples were incubated at 4°C overnight. Next, FITC-donkey anti-rabbit IgG (1:400; Jackson ImmunoResearch, Lancaster, PA, USA) was added, and the samples were incubated at room temperature for 60 min., followed by incubation with a goat anti-rat CD34 antibody (1:300; cat. no. ZDP0111041; R&D Systems, Minneapolis, MN, USA) at 4°C for 12 hrs, then a Cy3-donkey anti-goat IgG (1:400; Jackson ImmunoResearch) for 60 min. The cells were subsequently counter-stained by using DAPI and mounted with mounting medium. Throughout the above procedure, three PBS (pH = 7.4) washes were conducted after each step. To perform a semi-quantitative analysis of the obtained purity, 20 fields were randomly captured by using a fluorescence microscope (Olympus-IX51 with DP72-CCD; Olympus Corporation). The percentages of CD117+, CD34+, and both CD117+ and CD34+ cells were calculated.

### Induction of ARF

A set of 3-month-old female SD rats (*n* = 5 for each group) were anesthetized *via* intraperitoneal injection of sodium pentobarbital (30 mg/kg). Renal ischaemia–reperfusion was induced by bilateral clamping of the renal arteries for 45 min. Briefly, following abdominal incisions, the renal pedicles were bluntly dissected. For those mice subjected to ischaemia–reperfusion, microvascular clamps were used to clamp the bilateral renal pedicles for 45 min. Reperfusion commenced once the artery clamps were removed. Occlusion was verified visually based on a change in the colour of the kidneys to a paler shade and reperfusion based on blushing. One million renal TCs in PBS, 106 fibroblasts in PBS or PBS alone was injected into the caudal vein within 30 min. after reperfusion by using a 100-μl Hamilton syringe with a 30-gauge needle. During this procedure, the animals were kept well hydrated by using warm saline. The animals were killed at 48 hrs after reperfusion, and their kidneys were quickly removed and processed for histological evaluation, protein extraction and RNA extraction. Age- and weight-matched SD rats that were not subjected to any operation served as normal controls (*n* = 5), and rats subjected to sham operation without clamping of the renal arteries served as normal sham controls (*n* = 5) and were killed at the same time-point.

### Tracking of TCs *in vivo*

To track TCs *in vivo*, we generated GFP-expressing TCs (GFP-TCs) by using a lentivirus. To create stably transduced TCs expressing GFP, we packaged the lentiviral construct cDNA (Cat. # CD521A-1) into lentiviral pseudovirus particles according to the user manual provided by System Biosciences (SBI, Mountain View, CA, USA). Three days after infection, the GFP-TCs were analysed *via* FACS (BD, USA), and 48 hrs after injection of the TCs into the rats, we harvested kidney samples and generated frozen kidney sections (4 μm). The GFP-TCs were tracked *via* two-photon laser-confocal microscopy (Leica Microsystems Inc, LKB-II, Germany).

### Assessment of renal function

Renal functions were assessed by the levels of serum creatinine and BUN. Blood samples were obtained from orbit angular vein at 48 hrs after reperfusion. The blood was centrifuged (2600 × *g* for 10 min.) to isolate the serum.

### Histopathological evaluation of damage in the renal tubules

In addition to the above functional assessments, we evaluated the damage to the renal tubule *via* histopathology. For histopathological examination, kidney tissue was collected, sectioned coronally, fixed by using 10% formaldehyde and embedded in paraffin. Five-micrometre sections were generated and stained by using haematoxylin and eosin. The haematoxylin- and eosin-stained sections were semi-quantitatively graded at a 200× magnification for TID (tubular dilation and interstitial expansion with oedema inflammatory infiltrate) according to a scale of 0–3: a normal tubule-interstitium was scored as 0; mild TID affecting up to 25% of the field was scored as 1; moderate TID affecting 25–50% of the field was scored as 2; and severe TID exceeding 50% of the field was scored as 3. The examination was performed by two examiners who were blinded to the treatment group in 12 randomly selected consecutive fields, and a mean value was calculated for each kidney [41].

### Measurement of TEC proliferation in co-cultures with renal TCs *via* the CCK-8 assay and quantification of viable cells

To evaluate the influence of renal TCs on the proliferation of NRK-52E cells without direct contact, TCs and renal fibroblasts were layered onto the surfaces of porous membranes in the upper compartment of a Millicell TM system (Corning, Marlborough, MA, USA). The two cell populations were separated by 0.4-μm pore size transwell membranes, thus allowing subsequent recovery of soluble factors that were secreted without cell-to-cell contact [42].

To examine cell proliferation *via* the CCK-8 assay (Cell Proliferation and Cytotoxicity Assay Kit, Beyotime, Haimen, China), the renal cell population (2000 cells/well) was incubated in a 96-well plate beneath the membrane, and after 24 hrs, the system was treated with low-glucose DMEM without FBS. After co-culturing with TCs or renal fibroblasts for 12, 24, 48 or 72 hrs, a CCK-8 solution (10 μl) was added to each well, and the system was returned to the incubator at 37°C for 120 min. Cell proliferation was quantified according to the manufacturer's instructions.

The absorbance at 450 nm was measured by using a microtiter plate reader (ELX-800, Biotek, Winooski, VT, USA).

The percentage of viable cells was calculated relative to that of untreated control cells by using the following formula: survival rate (SR) = (mean absorbance of the test well/mean absorbance of the control) × 100%. The inhibition rate (IR) was calculated by using the formula IR = 100% − SR. The data were plotted as the means ± SD of three separate experiments, with four replicates for each experimental condition per experiment.

To count viable cells, we performed identical treatments, except for using 6-well plates (5 × 104 cells/well) rather than 96-well plates. At the time-points indicated in the figure legends, the Millicell TM inserts were removed, and the cells were washed with PBS and harvested by using a trypsin-EDTA solution. Proliferation was evaluated by counting the number of viable cells in the presence of Trypan blue.

### Analysis of TEC viability or apoptosis in co-cultures with renal TCs after ATP depletion

To evaluate cell viability *via* the CCK-8 assay, we incubated the renal cell population (5000 cells/well) in a 96-well plate beneath the transwell membrane by using high-glucose DMEM with 10% FBS. After 24 hrs, confluent NRK-52E cells were incubated in low-glucose DMEM in the presence of 2 μM antimycin A to block the mitochondrial respiratory chain at the level of complex III, thus avoiding oxidation of any substrate. In rat renal epithelial cells, antimycin A led to almost complete exhaustion of ATP stores after 45 min., with a slow and partial spontaneous recovery of the ATP levels observed following removal of the inhibitor [43]. After 2 hrs, the medium was removed, and the cells were washed three times with PBS, then incubated for 24 hrs at 37°C in low-glucose DMEM, either alone or in co-cultures with TCs or renal fibroblasts. The conditions of the CCK-8 assay were identical to those of the TEC proliferation assay.

Tubular epithelial cell apoptosis was determined *via* immunofluorescence staining for cleaved caspase-3. NRK-52E cells were seeded on sterile glass coverslips by using high-glucose DMEM with 10% FBS. The other treatments were identical to those performed in the CCK-8 assay. The immunofluorescence assay was performed as previously described by using a rabbit anti-rat cleaved caspase-3 primary antibody (1:400; Cell Signaling Technology, Danvers, MA, USA). Cell death was evaluated by counting the number of FITC-positive cells per field. For semi-quantitative analysis of purity, 20 fields were randomly captured by using a fluorescence microscope (Olympus-IX51 with DP72-CCD; Olympus Corporation).

### Quantitative real-time PCR analysis of inflammatory cytokines and growth factors *in vivo*

The mRNA levels of inflammatory cytokines and growth factors that are relevant to the regeneration of renal TECs were evaluated *via* quantitative real-time PCR in kidney tissues collected following the animal experiments (the role of growth factors in ARF). Total tissue RNA was extracted by using the TRIZOL reagent (Invitrogen™; Carlsbad, CA, USA) according to the manufacturer's instructions. The quality of the RNA was evaluated *via* gel electrophoresis, and the concentration of the RNA was measured based on the optical density at 260 nm. Reverse transcription-polymerase chain reaction (RT-PCR) was performed with an RT-PCR kit (Takara Bio Inc., Otsu, Shiga, Japan) in a 20-μl reaction system according to the manufacturer's instructions. The internal reference gene was β-actin. The PCR primers used in these assays are presented in Table1. The thermal cycling conditions included an initial incubation at 95°C for 30 sec., followed by 40 cycles at 95°C for 5 sec. and at 60°C for 34 sec. Real-time PCR was performed with the ABI Prism 7900 Sequencing Detection System (Applied Biosystems, Foster City, CA, USA).

**Table 1:** The sequence of gene-specific primer

Gene	The sequence of gene-specific primer
IL-1β	F: 5′-GCTGACAGACCCCAAAAGAT-3′
	R: 5′-TGTCGAGATGCTGCTGTGAG-3′
IL-6	F: 5′-GTTGCCTTCTTGGGACTGAT-3′
	R: 5′-TGAAGTCTCCTCTCCGGACT-3′
TNF-α	F: 5′-CCTTATCTACTCCCAGGTTCTC-3′
	R: 5′-AGGGGCCATCCACAGTCTTC-3′
IL-10	F: 5′-TGCAACAGCTCAGCGCA-3′
	R: 5′-GTCACAGCTTTCGAGAGACTGGAA-3′
HGF	F: 5′-TATTTACGGCTGGGGCTACA-3′
	R: 5′-ACGACCAGGAACAATGACAC-3′
TGF-α	F: 5′-AAGGGCAAGAGGACAAGAGAG-3′
	R: 5′-ACGAGGAGGCTAATCCAACTTA-3′
TGF-β	F: 5′-CTTGCCCTCTACAACCAACA-3′
	R: 5′-CTTGCGACCCACGTAGTAGA-3′
EGF	F: 5′-TGATTGAAATGGCCGATCTA-3′
	R: 5′-AACCACACGTGATCCTCAAA-3′
bFGF	F: 5′-AACGCCTGGAGTCCAATAAC-3′
	R: 5′-ATACTGCCCAGTTCGTTTCA-3′
PDGF	F: 5′-AGTCGAGTCGGAAAGCTCAT-3′
	R: 5′-ACACCTCTGTACGCGTCTTG-3′
IGF-1	F: 5′-GGTGGACGCTCTTCAGTTCG-3′
	R: 5′-AGTACATCTCCAGCCTCCTCAG-3′
β-actin	F: 5′-TGACCCAGATCATGTTTGAGA-3′
	R: 5′-GGCATACAGGGACAACACAG-3′

### Quantitative real-time PCR analysis of growth factors *in vitro*

We evaluated the paracrine effects of growth factors in TCs under two conditions. First, we employed the same co-culture system used in the CCK-8 assay, as described above. TCs, renal fibroblasts and NRK-52E cells were used to assess the mRNA levels of certain cytokines. Second, we mimicked RIR *in vitro* through stimulation with inflammatory cytokines. We incubated TCs and renal fibroblasts (5 × 105 cells/well) in 6-well plates for 24 hrs in medium containing 10% FBS. After stimulation for 48 hrs with recombinant rat tumour necrosis factor-α (TNF-α; 20 ng/ml), recombinant rat IL-1b (2 ng/ml), recombinant rat interferon-γ (IFN-γ; 250 U/ml), lipopolysaccharide (LPS; 100 mg/ml) and phorbol myristate acetate (PMA; 100 ng/ml), the cells were harvested to measure the mRNA levels of certain cytokines. The quantitative real-time PCR assays were performed as previously described.

### Western blot analysis

Cell lysates were prepared, and the cytoplasmic protein fraction was isolated from 20 to 40 μg of kidney tissue at 4°C. Equal amounts of proteins were separated *via* SDS–PAGE and transferred to PVDF membranes. Next, primary antibodies, including anti-caspase-3 (1:500; Santa Cruz, Santa Cruz, CA, USA), anti-pNF-κB (1:1000; Cell Signaling Technology), anti-NF-κB (1:1000; Cell Signaling Technology), were applied, followed by incubation at 37°C for 2 hrs with gentle shaking and subsequent incubation for 1 hr in peroxidase-conjugated secondary antibodies (Jackson ImmunoResearch) at room temperature. The immunoreactive bands were visualized by using an ECL system (Amersham Pharmacia, Piscataway, NJ, USA). To control for lane loading, the same membranes were also probed by using an anti-β-actin antibody (Abcam, Cambridge, UK) according to the molecular weight of the target proteins. The signals were quantified *via* scanning densitometry by using a Bio-Image Analysis System (Bio-rad, Hercules, CA, USA). The results from each experimental group were expressed as the integrated intensity relative to that of the control sample measured within the same trial.

### Statistical analysis

The data are expressed as the means ± SD based on at least three independent experiments. The difference between the groups was determined *via* one-way anova, and comparisons between two groups were performed with Student's *t*-test by using SPSS 11.0 software (IBM, Armonk, NY, USA). A value of *P* < 0.05 was considered statistically significant.

## Results

### Identification of renal TCs

**Figure 1:**
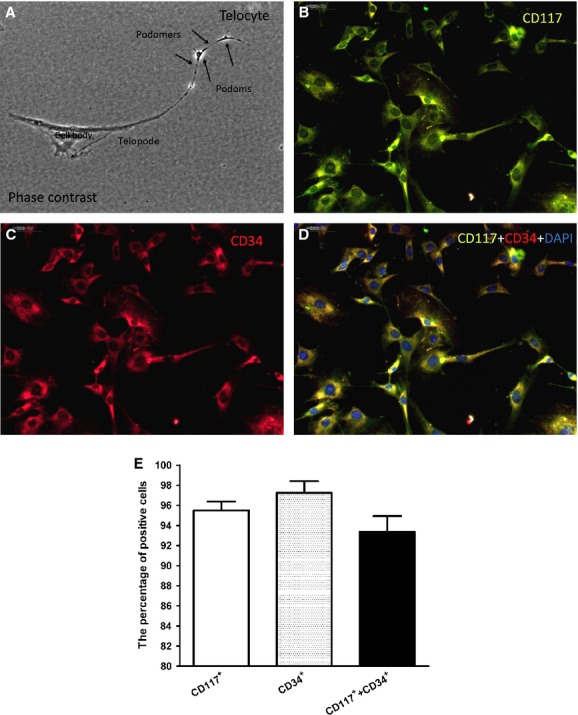
Purity of the isolated renal telocytes (TCs). Phase-contrast microscopy of kidney TCs in primary culture (A). Note the typical, very long telopodes (more than 40 μm). The unique structure of the telopodes is also apparent, consisting of alternating dilations (podoms) and thin segments (podomers). Direct magnification: 400 × . Double immunofluorescence staining against CD117 and CD34 combined with cell counting revealed that ∽95.5 ± 0.01% of the cells were c-kit-positive (B), while ∽97.5 ± 0.02% were CD34-positive (C), and ∽93.5 ± 0.05% were both c-kit- and CD34-positive (D). B: Anti-CD117 (green); C: anti-CD34 (red); D: merged images of CD117, CD34 and DAPI staining. (E) Quantification of cells that were positive for CD117, CD34, and both CD117 and CD34 (n = 5).

Based on phase-contrast microscopy, the primary culture of isolated CD117+ renal TCs displayed renal TCs with piriform/spindle/triangular cell bodies containing long, slender Tps, showing an alternation of thick segments (podoms) and thin segments (podomers; Fig.1A). The renal TCs with this unique morphology were positive for both CD117 and CD34 (Fig.1B–D). The purity of the isolated renal TCs was determined based on double immunofluorescence staining of CD117 and CD34. Approximately 95.50 ± 1.76% of the total cells were CD117-positive, while approximately 97.25 ± 2.33% were CD34-positive and ∽93.38 ± 3.11% were both CD117- and CD34-positive (Fig.1E and Table2).

**Table 2:** Quantification of cells that were positive for CD117, CD34 and CD117 and CD34

Staining	Percentage (%)
CD117	95.50 ± 1.76
CD34	97.25 ± 2.33
CD117&CD34	93.38 ± 3.11

### TCs were detected histologically in damaged kidneys

**Figure 2:**
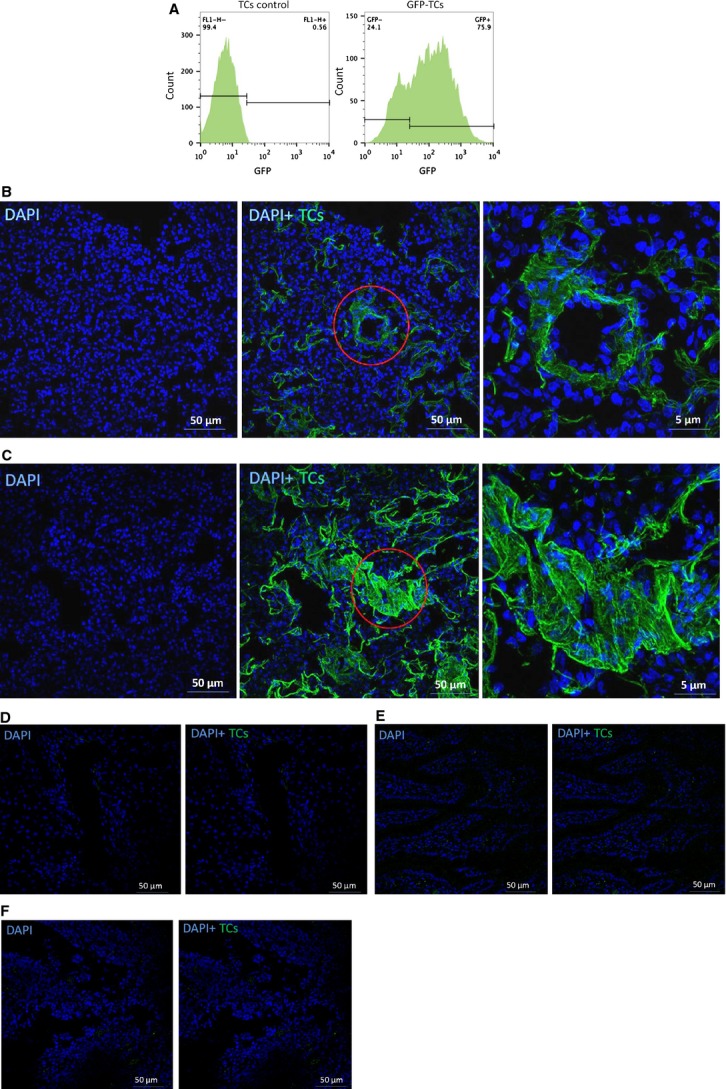
Tracking of renal telocytes (TCs) following renal ischaemia–reperfusion injury. Three days after infection, ∽75.9% of the TCs were GFP positive (A). Confocal microscopy revealed that GFP-TCs were present in the kidneys (B) and the lungs (C). However, no TCs were detected in the liver (D), the spleen (E) or the intestine (F).

To further evaluate the renal delivery of intravenously infused TCs, we generated GFP-TCs. The FACS results revealed that ∽75.9% of the TCs were GFP-positive (Fig.2A). Frozen sections showed GFP expression in both the kidney and the lungs following the injection of GFP-TCs (Fig.2B and C). The TCs in the kidney displayed piriform/spindle/triangular cell bodies that primarily congregated around the vessel. The number of TCs in the kidney was slightly lower than in the lungs. Moreover, there were almost no TCs found in the liver, the spleen or the intestine (Fig.2D–F).

### Transplantation of renal TCs attenuates renal dysfunction induced by IRI

**Figure 3:**
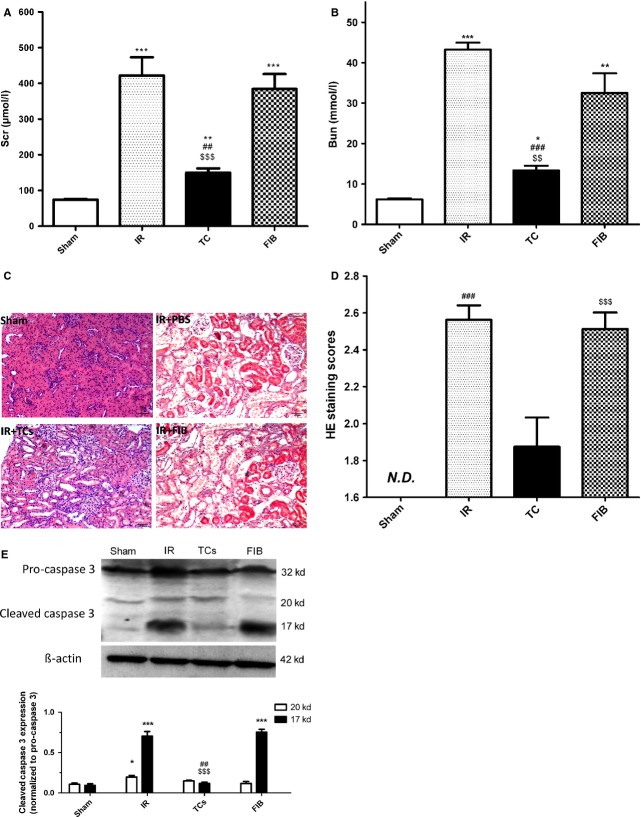
Effects of renal telocytes (TCs) on renal function, histological changes and the expression of cleaved caspase-3 at 48 hrs after renal ischaemia–reperfusion injury (IRI). Scr (A) and BUN (B) levels were increased following renal IRI. Renal TC transplantation prevented renal dysfunction, while FIB injection had no effect after renal IRI. The histological damage (C) following IRI was also ameliorated by TC injection based on a semi-quantitative assessment (D). The expression of cleaved caspase-3 was decreased after renal TC transplantation compared with the expression in the PBS injected and FIB injected groups (E). * Statistically significant difference compared with the sham group; # statistically significant difference between the TC-injected group and the PBS-injected group; $ statistically significant difference between the TC-injected group and the FIB-injected group. *P < 0.05, **P < 0.01, ***P < 0.001.

The rats subjected to renal IRI exhibited a significant increase in Scr (421.75 ± 102.81 μmol/l) and BUN (43.25 ± 3.40 mmol/l) levels compared with the sham-operated rats (51.50 ± 11.47 μmol/l and 5.20 ± 0.43 mmol/l respectively). Transplantation of renal TCs decreased Scr and BUN levels, while transplantation of renal fibroblasts had no such effect (Fig.3A and B, Table3). Following IRI, TEC necrosis and tubular injuries were clear (Fig.2C). These injuries included loss of the brush border and dilation of the renal tubules and urinary cylinder. As previously mentioned, the assessment of histological injury was performed with a scoring system ranging from 0 to 3 points. The TC-injected groups presented a lower score compared with the fibroblast-injected and PBS-injected groups (Fig.3D and Table4). Renal TC transplantation also resulted in reduced expression of cleaved caspase-3 compared with the injection of both renal fibroblasts and PBS (Fig.3E), which suggests that renal TCs could inhibit TEC apoptosis after IRI.

**Table 3:** Effects of renal TCs on renal function (Scr& Bun)

Treatment	Scr (μmol/l)	Bun (mmol/l)
Sham	51.50 ± 11.47	5.20 ± 0.43
IR with PBS	421.75 ± 102.81*	43.25 ± 3.40*
IR with TCs	110.25 ± 19.92*,#,$	8.33 ± 1.71*,#,$
IR with FIB	384.50 ± 83.43*	32.50 ± 9.75*

* Statistical difference with respect to Sham group (*P* < 0.05);

# Statistical difference between TCs injection group and PBS injection group (*P* < 0.05);

$ Statistical difference between TCs injection group and FIB injection group (*P* < 0.05).

**Table 4:** Histological injury score

Treatment	Score
Sham	N/A
IR with PBS	2.56 ± 0.16*
IR with TCs	1.88 ± 0.32
IR with FIB	2.51 ± 0.18*

* Statistical difference with respect to IR with TCs (*P* < 0.05).

### TCs protect against IRI through inflammation-independent mechanisms

**Figure 4:**
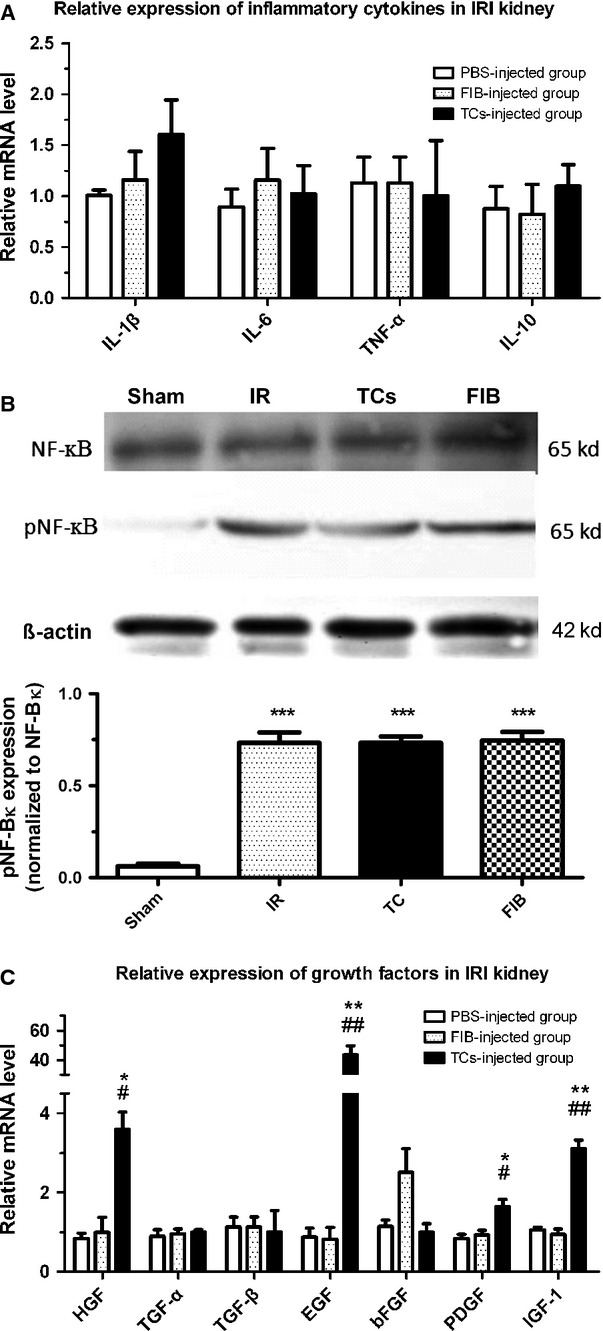
Effects of renal telocytes (TCs) on the inflammatory response and the levels of growth factors. Ischaemia–reperfusion injury (IRI) induced an up-regulation of pro-inflammatory cytokines that did not differ between any of the animal groups (A). The NF-κB signalling pathway was activated after IRI (B), which contributed to the pro-inflammatory response. The mRNA levels of HGF, EGF, PDGF and IGF-1 were significantly increased in the TC-injected group compared with the renal fibroblast–injected group and the PBS-injected group (C). *Statistically significant difference compared with the PBS-injected group; #statistically significant difference between the TC-injected group and the FIB-injected group. *P < 0.05, **P < 0.01, ***P < 0.001.

High mRNA levels of pro-inflammatory cytokines, including IL-1 and TNF-α, were detected in all of the animal groups (Fig.4A). Consequently, we evaluated the phosphorylation level of NF-κB. A significant increase in the phosphorylation level of NF-κB was detected after IRI with or without TC injection (Fig.4B), while the expression of NF-κB was not affected. On the other hand, the mRNA levels of HGF, EGF, PDGF and IGF-1 were significantly increased in the TC-injected group compared with the renal fibroblast–injected and PBS-injected groups (Fig.4C). These results reveal that the protective effect of TCs on IRI may not rely on an anti-inflammatory mechanism, but rather on the secretion of growth factors.

### TCs have no effect on renal TEC proliferation or apoptosis following ATP depletion *in vitro*

**Figure 5:**
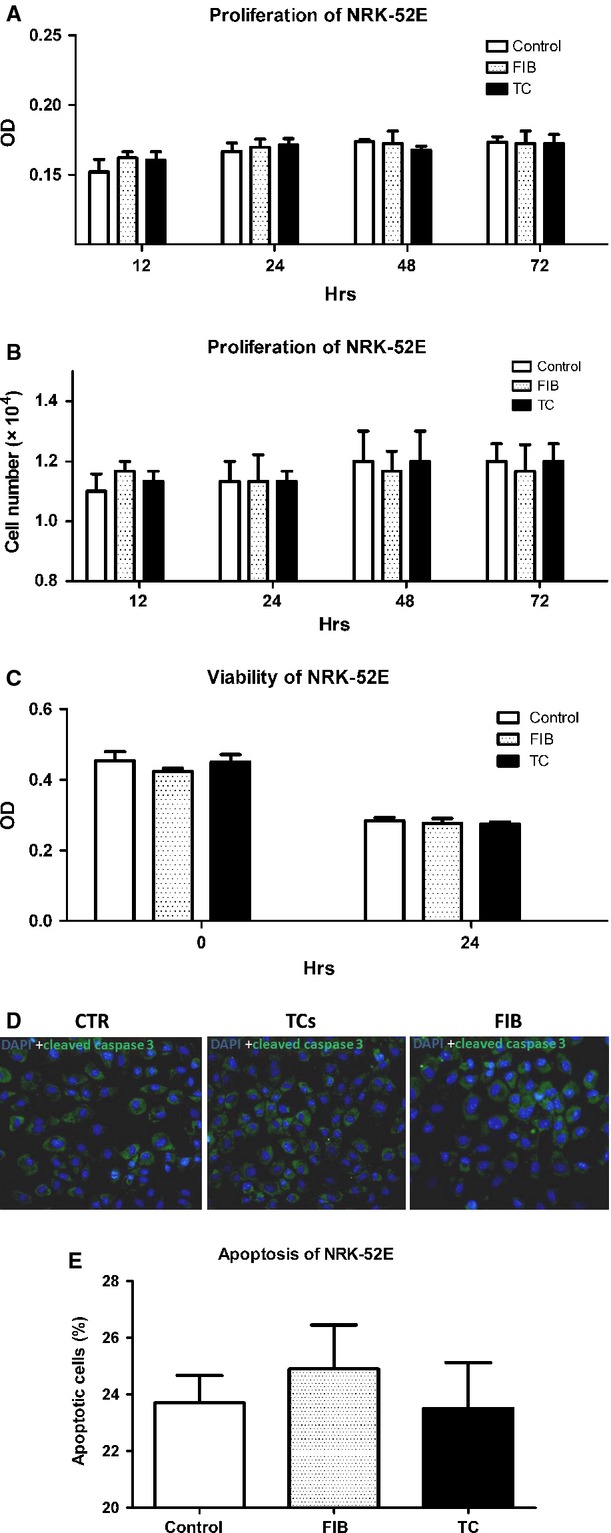
Telocytes (TCs) did not exert any effect on renal TEC proliferation or apoptosis following ATP depletion *in vitro*. Neither TCs nor renal fibroblasts stimulated the proliferation of NRK-52E cells in a physically separated co-culture system (A and B). NRK-52E cells were co-cultured with renal TCs or FIBs in a MillicellTM system for the time periods indicated on the *x*-axis, as described in the Materials and Methods section. Proliferation was evaluated *via* the CCK-8 assay and through counting the number of viable cells in the culture. (B) ATP depletion insult caused reduced cell viability and an increase in the cell death rate based on the CCK-8 assay and cleaved caspase-3 immunofluorescence staining. The expression of cleaved caspase-3 was evaluated through semi-quantitative assessment (C–E). CTR: control (renal cells cultured without TCs or FIBs).FIB (renal cells cultured with FIBs). TCs(renal cells cultured with TCs).

Cultures were examined at 12, 24, 48 and 72 hrs to determine whether TCs or renal fibroblasts were able to stimulate the proliferation of NRK-52E cells in the MillicellTM system under co-culture conditions. After a 72-hr lag phase, neither the TCs nor renal fibroblasts had induced the proliferation of NRK-52E cells in FBS-free medium (Fig.5A and B, Table5). We evaluated whether co-culture with TCs or renal fibroblasts prevented NRK-52E cell death. ATP depletion is one component of ischaemic insult. To mimic this condition *in vitro*, NRK-52E cells were incubated in antimycin A for 2 hrs, followed by incubation for a further 24-hr period after removal of the inhibitor. The ATP depletion insult led to reduced cell viability and an increase in the cell death rate based on the CCK-8 assay and cleaved caspase-3 immunostaining (Fig.5C–E, Tables6 and 7). Co-culture with TCs or renal fibroblasts did not prevent TEC death after ATP depletion.

**Table 5:** Proliferation of NRK-52E

Treatment	Hours			
	12 h	24 h	48 h	72 h
Control				
OD	0.15 ± 0.02	0.17 ± 0.01	0.17 ± 0.00	0.17 ± 0.01
Number (10^4^)	1.10 ± 0.10	1.13 ± 0.12	1.20 ± 0.17	1.20 ± 0.10
FBI				
OD	0.16 ± 0.01	0.17 ± 0.01	0.17 ± 0.02	0.17 ± 0.02
Number (10^4^)	1.17 ± 0.06	1.13 ± 0.15	1.17 ± 0.12	1.17 ± 0.15
TC				
OD	0.16 ± 0.01	0.17 ± 0.01	0.17 ± 0.00	0.17 ± 0.01
Number (10^4^)	1.13 ± 0.06	1.13 ± 0.06	1.20 ± 0.17	1.20 ± 0.10

Control: NRK-52E culture in FBS-free medium;

FIB: NRK-52E co-culture with renal fibroblasts in FBS-free medium;

TC: NRK-52E co-culture with renal telocytes in FBS-free medium.

**Table 6:** Viability of NRK-52E

Treatment	Hours	
	0 h	24 h
	OD	
Control	0.45 ± 0.05	0.28 ± 0.02
FIB	0.42 ± 0.02	0.28 ± 0.02
TC	0.45 ± 0.04	0.27 ± 0.01

Control: NRK-52E culture in FBS-free medium with antimycin A;

FIB: NRK-52E co-culture with renal fibroblasts in FBS-free medium with antimycin A;

TC: NRK-52E co-culture with renal telocytes in FBS-free medium with antimycin A;0 h:cell culture in high-glucose DMEM with 10% FBS;

24 h: 24 hours after 2 h-antimycin treatment.

**Table 7:** Apoptosis of NRK-52E

Treatment	Apoptotic cells (%)
Control	23.70 ± 1.94
FIB	24.90 ± 3.10
TC	23.50 ± 3.25

Control: NRK-52E culture in FBS-free medium with antimycin A;

FIB: NRK-52E co-culture with renal fibroblasts in FBS-free medium with antimycin A;

TC: NRK-52E co-culture with renal telocytes in FBS-free medium with antimycin A.

### TCs display no advantage in paracrine growth factor expression *in vitro* compared with renal fibroblasts

**Figure 6:**
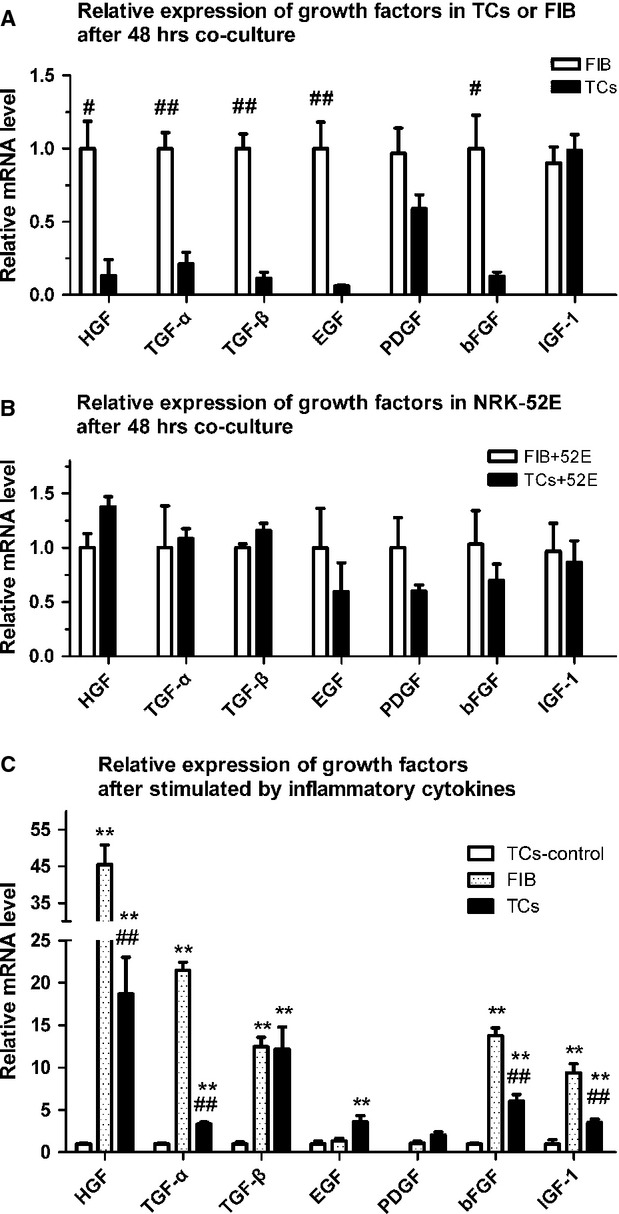
Paracrine effects of growth factors between telocytes (TCs) and renal fibroblasts under two conditions. The mRNA expression of HGF, TGF-α, TGF-β and EGF was significantly lower in TCs in renal fibroblasts in the co-culture system identical to that of the CCK-8 assay (A). There was no significant difference in the mRNA expression levels of the growth factors between the NRK-52E cells co-cultured with TCs and renal fibroblasts (B). After stimulation of TCs by using inflammatory cytokines, the mRNA expression levels of HGF, TGF-α, TGF-β, EGF, bFGF and IGF-1 were significantly higher than in non-stimulated TCs. However, the mRNA expression levels of HGF, TGF-α, bFGF and IGF-1 were significantly lower in TCs than that in renal fibroblasts following stimulation by using inflammatory cytokines (C). TC-control group: TCs cultured with complete medium. *Statistically significant difference compared with the TC-control group; #statistically significant difference between the TC and FIB groups. *P < 0.05, **P < 0.01, ***P < 0.001.

We compared the paracrine effects of growth factors between TCs and renal fibroblasts under two conditions. In the co-culture system, the mRNA expression of HGF, TGF-α, TGF-β and EGF was significantly lower in the TCs than in the renal fibroblasts (Fig.6A). However, there were no significant differences in mRNA expression levels between the NRK-52E cells co-cultured with TCs and those co-cultured with renal fibroblasts (Fig.6B). Under conditions mimicking kidney IRI *via* stimulation with inflammatory cytokines, the mRNA expression of HGF, TGF-α, bFGF and IGF-1 was also significantly lower in the TCs than in the renal fibroblasts (Fig.6C).

## Discussion

Telocytes have been detected in many organs [5–36], and we recently identified renal TCs in the renal cortex [17]. However, the biological function of these cells remains unknown. TCs have been reported to form a 3D network throughout the organ interstitium that communicates with surrounding organ-specific structures, immune cells, nerve endings and even stem cells [6,8,9,11,12]. Telocytes may participate in neo-angiogenesis during the late stage of myocardial infarction *via* direct (physical) contact with the endothelial tubes as well as *via* an indirect (chemical) positive influence in ‘angiogenic zones’ [10]. Simultaneous transplantation of cardiac TCs in infarcted zones of the heart was shown to decrease the infarct size and improve myocardial function [44]. Gene expression profiling revealed that some genes that are highly expressed in TCs are related to components or regulators of the basement membrane [38].

Based on our results, we first revealed that renal TCs exhibited the ability to promote repair after ischaemic renal tubular injury, similar to recently reported findings regarding the ischaemic heart [44]. Furthermore, we demonstrated that injection of renal TCs can attenuate renal dysfunction and ameliorate renal histological damage following renal IRI.

Inflammation and necrosis have been shown to be the principal pathophysiological alterations that occur during renal IRI [45–47]. The direct damage to renal function is as a result of the apoptosis of TECs [48–51]. Mesenchymal stem cells (MSCs) have a strong therapeutic effect on renal IRI because of their immunomodulatory and anti-apoptotic effects, rather than their differentiation into target cells [52]. NF-κB is an important downstream effector of the innate immune signalling pathway and is also involved in a vital inflammatory cascade following renal IRI. The activation/phosphorylation and nuclear translocation of NF-κB lead to an enhanced immunoinflammatory response. In turn, increased levels of pro-inflammatory cytokines, including TNF-α and IL-1β, promote the phosphorylation of NF-κB [53]. We discovered that renal TCs failed to suppress the activation of the NF-κB signalling pathway; TCs did not decrease the phosphorylation level of NF-κB or IκB following IRI. Consequently, the mRNA levels of pro-inflammatory cytokines, such as IL-1 and TNF-α, were up-regulated. Therefore, unlike MSCs, TCs exert no anti-inflammatory effect on renal IRI [52].

Several growth factors, including HGF, EGF, IGF-1, TGF-α and TGF-β, are produced in the kidneys and function as autocrine or paracrine regulators of renal IRI. They play an important role in TEC proliferation and protection against apoptosis [54]. We detected significantly increased mRNA levels of HGF, EGF, PDGF and IGF-1 in TC-injected kidneys, which may be either a direct or secondary (*via* a principal reduction of kidney injury) result of this treatment.

We also examined whether TCs could have a similar effect on TECs *in vitro*. However, in FBS-free medium, TCs were not able to induce the proliferation of TECs. In addition, under ATP depletion conditions, TCs could not prevent TEC from death. A comparison of the paracrine effect of growth factors between TCs and renal fibroblasts in FBS-free and inflammatory cytokine–containing medium indicated that TCs did not respond differently to paracrine growth factors compared with renal fibroblasts. Moreover, there was no significant difference in the mRNA expression of growth factors between TECs co-cultured with TCs versus renal fibroblasts.

In a previous study, by using transmission electron microscopy, we revealed that renal TCs were located around tubules and vessels, with their Tps surrounding the endothelium or the basement membrane [17]. Our current results showed that renal TCs were able to protect TECs only *in vivo* and could not directly protect them *in vitro*. Following the transplantation of TCs into IRI kidneys, TCs congregated in the kidney around vessels. However, more TCs were found in the lungs. One explanation for this result is that TCs are trapped within the pulmonary capillaries, preventing access to other organs, as the size of the cells is typically larger than that of the pulmonary capillaries [55]. Following IRI, as a result of activation of the innate and adaptive immune systems, many inflammatory cells congregate in the kidneys and secrete chemokines that can guide TCs to the injured kidney [52]. Based on the low number of TCs found in the kidneys, we can infer that there is no sufficient amount of TCs to differentiate into TECs. Therefore, we can essentially exclude the possibility that TCs acted as stem cells to improve renal function *via* a differentiation-dependent mechanism. Some studies on the heart have indicated that TCs might act as nurse cells supporting cardiac stem cells, contributing to both cardiac cellular homeostasis and endogenous repair/remodelling after injury [56]. Therefore, alternative mechanisms should also be considered. For example, TCs in the kidney and the heart might function in a similar way. It is assumed that TCs contribute to repair following renal IRI by supporting other cells, such as MSCs and tubular stem cells, balancing the tubular basement membrane to protect TECs. However, the exact underlying mechanism remains unknown, and further studies are therefore required.

It might be interesting to consider that TCs network could act as a local primitive nervous system [57], or TCs could be nurse cells in the architectural organization of engineered heart tissue [58].

## Conclusion

Overall, our data demonstrated, for the first time, that renal TCs isolated from the kidney cortex can protect against renal IRI, although the underlying mechanism remains unclear. Our findings indicated that renal TCs cannot differentiate into TECs or suppress the inflammatory response following renal IRI. Instead, the mechanism of their protective effect may depend on growth factors.
